# Associations of B Vitamin-Related Dietary Pattern during Pregnancy with Birth Outcomes: A Population-Based Study in Northwest China

**DOI:** 10.3390/nu14030600

**Published:** 2022-01-29

**Authors:** Shanshan Li, Danmeng Liu, Yijun Kang, Pengfei Qu, Baibing Mi, Zhonghai Zhu, Lixin Han, Yaling Zhao, Fangyao Chen, Leilei Pei, Lingxia Zeng, Duolao Wang, Hong Yan, Shaonong Dang

**Affiliations:** 1 School of Public Health, Shandong First Medical University & Shandong Academy of Medical Sciences, Taian 271016, China; lishanshan@sdfmu.edu.cn; 2 Department of Epidemiology and Health Statistics, Health Science Center, Xi’an Jiaotong University, Xi’an 710061, China; liudanmeng1214@stu.xjtu.edu.cn (D.L.); tjkyj@xjtu.edu.cn (Y.K.); xjtu.mi@xjtu.edu.cn (B.M.); zhonghai_zhu@xjtu.edu.cn (Z.Z.); zhaoyl666@xjtu.edu.cn (Y.Z.); chenfy@xjtu.edu.cn (F.C.); peileilei424@163.com (L.P.); tjzlx@xjtu.edu.cn (L.Z.); yanhonge@xjtu.edu.cn (H.Y.); 3 Translational Medicine Center, Northwest Women’s and Children’s Hospital, Xi’an 710061, China; xinxi3057@stu.xjtu.edu.cn; 4 Key Laboratory of Trace Elements and Endemic Diseases of National Health Commission, Health Science Center, Xi’an Jiaotong University, Xi’an 710061, China; weijianweijikongchu@shaanxi.gov.cn; 5 Disease Control and Prevention Division, Shaanxi Provincial Health Commission, Xi’an 710000, China; 6 Department of Clinical Sciences, Liverpool School of Tropical Medicine, Liverpool L3 5QA, UK; duolao.wang@lstmed.ac.uk; 7 Nutrition and Food Safety Engineering Research Center of Shaanxi Province, Xi’an 710061, China

**Keywords:** dietary pattern, B vitamins, pregnancy, birth weight, small-for-gestational-age, reduced rank regression

## Abstract

This study aimed to derive a maternal dietary pattern to explain the variation in B vitamins during pregnancy and to investigate this pattern in relation to birth outcomes. A total of 7347 women who gave birth to live newborns less than one year were included. Their dietary pattern during pregnancy was derived using the reduced-rank regression method with six B vitamins as response variables. Associations between dietary pattern score and birth weight, gestational age at delivery, birth weight Z score, low birth weight, preterm, and small-for-gestational-age (SGA) were estimated using generalised linear mixed models. We identified a high B-vitamin dietary pattern characterised by high intakes of animal foods, vegetables, fungi and algae, legumes, and low intakes of oils and cereals. Women in the highest quartile of this pattern score had newborns with a 44.5 g (95% CI: 13.8, 75.2 g) higher birth weight, 0.101 (95% CI: 0.029, 0.172) higher birth weight Z score, and 27.2% (OR: 0.728; 95% CI: 0.582, 0.910) lower risk of SGA than those in the lowest quartile. Our study suggested that adherence to the high B-vitamin dietary pattern during pregnancy was associated with a higher birth weight and a lower risk of SGA.

## 1. Introduction

Birth outcomes, which commonly refer to birth weight and gestational age, are not only closely related to the morbidity and mortality of infants and young children [1,2], but are also key predictors of chronic non-communicable diseases in adulthood [3,4,5]. As the economy grows by leaps and bounds, the health status of women and children in China has been greatly improved in the past few decades [6]. However, China has the largest population in the world, of more than 1.4 billion. According to the latest WHO data, China ranks among the top five countries worldwide in terms of the number of adverse birth outcomes such as preterm, low birth weight (LBW), and small-for-gestational-age (SGA) [7,8,9].

The aetiology of most types of adverse birth outcomes is complex. As a modifiable risk factor, maternal nutrition during pregnancy has been widely discussed for its potential to prevent adverse birth outcomes [10]. B vitamins are a class of water-soluble micronutrients that act as co-enzymes in numerous catabolic and anabolic enzymatic reactions [11]. To our knowledge, the associations of other B vitamins than folate (vitamin B9) with adverse birth outcomes remain unclear. Some epidemiologic studies have reported that maternal dietary intakes of B vitamins are inversely related to the risk of adverse birth outcomes [12,13,14]. Since B vitamins share similarities in food sources and biological functions, these studies failed to clarify the individual effect of specific B vitamins, nor did they capture the cumulative effect of multiple B vitamins [15].

Recent nutritional epidemiological studies have shifted the focus from single nutrients to dietary patterns, which describe the overall diet and better reflect the interactive or synergistic effects of different nutrients [16]. Of note, Hoffmann et al. proposed the reduced rank regression (RRR) method, by which dietary patterns are derived using a posteriori statistical analysis, but response variables (e.g., nutrients) are chosen using a priori knowledge that suggests these variables are hypothesized to be related to health outcomes [17]. The RRR method identifies the linear combination of food groups that account for as much variation as possible in a set of response variables. Compared to traditional methods (e.g., Health Eating Index and principal component analysis), the RRR method has the advantage of building biological pathways through which diet affects health outcomes [18].

Therefore, based on the cross-sectional data in northwest China, we aimed to identify a maternal dietary pattern that maximally explains the variation in B vitamins using the RRR method. Furthermore, we sought to assess the associations of this pattern with birth outcomes after controlling for covariates.

## 2. Materials and Methods

### 2.1. Study Design and Participants

The present study used data from a cross-sectional study conducted in Shaanxi Province of northwest China. The design and methodology of the cross-sectional study have been described previously in detail [19,20]. Briefly, 30,027 women who were pregnant during 2010–2013 and had pregnancy outcomes were selected using the stratified multi-stage random sampling method. First, twenty counties and ten districts were randomly selected from the Shaanxi Province according to the proportion of urban to rural population, population density and fertility rate. Second, six townships and three streets were randomly selected from the sampled counties and districts, separately. Third, six villages and communities were randomly selected from the sampled townships and streets, separately. Finally, thirty and sixty women were randomly selected from the sampled villages and communities, separately. All participants were interviewed from August to November 2013. Information on maternal general and pregnancy characteristics, and neonatal outcomes were collected by a standardised and structured questionnaire. Furthermore, 7750 women who gave birth to live newborns less than one year ago were interviewed to obtain information on dietary intakes during pregnancy. For this study, 403 women were excluded due to multiple births (*n* = 87) or implausible energy intake (<4500 or >20000 kJ/d) (*n* = 316) [21]. Consequently, a total of 7347 women were included in the final analysis, with a median of 3 months (10–90th percentiles: 0–7 months) after delivery. A flow chart of the selection of study participants is displayed in Figure S1.

### 2.2. Dietary Assessment

The dietary intakes during pregnancy were estimated retrospectively using a semi-quantitative food frequency questionnaire (FFQ). Since dietary intakes did not change to a great extent throughout pregnancy [22,23,24], and it was cumbersome to collect dietary data for different trimesters, we assessed the average dietary intakes over the whole pregnancy at one time. The FFQ applied in this study was established based on a validated FFQ designed for pregnant women during the third trimester in northwest China [25]. The validation study showed good correlations between nutrients estimated by the FFQ and six repeated 24 h recalls. Pearson’s correlation coefficients ranged from 0.53 for cholesterol and carotene, to 0.70 for vitamin E and potassium [25]. The FFQ used in this study consisted of 107 items. For the five food items concerning edible oil and condiments, the weight consumed per month and the number of family members were recorded. For the other 102 food items, consumption frequencies were assessed on an eight-level scale (never or almost not, less than one time/month, one to three times/month, one time/week, two to four times/week, five to six times/week, one time/day, or more than two times/day), and portion sizes were estimated on a three-level scale (large, medium, or small) using the food photographs [26]. The daily intakes of total energy and nutrients were estimated by multiplying consumption frequency by the portion size of each food item and their corresponding nutrient content abstracted from the China Food Composition Table [27,28]. All nutrient intakes were adjusted for total energy intake with the use of the residual method [29].

### 2.3. Birth Outcomes

Neonatal outcomes, including sex, gestational age at birth, and birth weight were obtained from the Medical Certificate of Birth. Birth weight was measured to the nearest 10 g. Gestational age at birth was the number of weeks from the first day of the last menstrual period to the date of delivery. The sex- and gestational age-adjusted birth weight Z score was calculated based on the International Fetal and Newborn Growth Consortium for the 21st Century (INTERGROWTH-21st) standards [30]. LBW referred to a birth weight less than 2500 g [31]. Preterm was defined as gestational age at birth less than 37 weeks [32]. SGA referred to a birth weight Z score below the 10th percentile [33].

### 2.4. Covariates

The covariates selected based on previous studies can be categorised as maternal socio-demographic characteristics and health-related behaviours during pregnancy [34,35,36]. Socio-demographic characteristics comprised geographic area, residence, age at delivery, education, occupation, household wealth index, and parity. The principal component analysis was used to construct the household wealth index based on household income and expenditure, type of house, number of appliances, and number of vehicles [37]. Poor was defined as the first principal component below the 33.3rd percentile. Health-related behaviours included smoking, alcohol consumption, pregnancy complications, medication use, as well as folic acid, iron, calcium, and multivitamin supplementation. Only passive smoking was considered as a covariate because of pregnant women’s low prevalence of active smoking in our study areas. Passive smoking was defined as a non-smoker being exposed to tobacco smoke for at least 15 min per day. Self-reported pregnancy complications consisted of anaemia, hypertension, diabetes, intrahepatic cholestasis, and so on.

### 2.5. Statistical Analysis

Maternal dietary pattern during pregnancy was obtained by the reduced rank regression (RRR) method using the PLS procedure in SAS [17]. Before conducting this analysis, 107 food items were categorised into 21 food groups according to their similarities in nutrient content and culinary usage (Table S1). The predictor variables were the daily intakes of 21 food groups in grams, while the response variables were the daily intakes of thiamin (vitamin B1), riboflavin (vitamin B2), niacin (vitamin B3), vitamin B6, folate, and vitamin B12 in grams. The number of RRR factors derived was equal to the number of response variables. Considering that the first RRR factor explained the highest proportion of the variation in response variables, only this factor was considered as the dietary pattern of interest [18,38]. Food groups with an absolute factor loading ≥ 0.2 were utilised to characterise the dietary pattern. The dietary pattern score of each participant was outputted by summing up the intakes of each food group and multiplying with the corresponding factor loadings and categorised into quartiles. Pearson’s correlation coefficients and 95% CIs between the dietary pattern score and each response variable were calculated.

Binary and continuous variables were presented as *n* (%) and means ± SDs, respectively. Linear trends across the quartiles of dietary pattern score were estimated using Cochran-Armitage tests for maternal characteristics and univariate linear regression models for nutrient intakes. Given the significant within-group homogeneity for most birth outcomes at the county level, the generalised linear mixed model with a random intercept at the county level was employed to estimate the associations of maternal dietary pattern score with birth outcomes. The dietary pattern score was analysed both as a continuous variable (per 1-SD increase) and a categorised variable (the lowest quartile as the reference). To assess the robustness of these associations, a total of three models were constructed in sequence. Model 1 included no covariates. Model 2 included socio-demographic characteristics. Model 3 included all covariates in Model 2 plus health-related behaviours. The link functions of continuous and binary dependent variables were “identity” and “logit”, separately. Linear trends for birth outcomes across the quartiles of dietary pattern score were assessed by putting the quartile number into models as an ordinal variable. Since multivitamins usually contain B vitamins, stratified analyses by folic acid and multivitamin supplementation were performed to test whether these supplements modified the associations of dietary pattern score with birth outcomes. Potential effect modifications were examined by including interaction terms in the fully adjusted models.

All statistical analyses were performed using SAS software (version 9.4; SAS Institute Inc., Cary, CA, USA). A two-sided *p* < 0.05 was considered statistically significant.

## 3. Results

### 3.1. Dietary Pattern

A total of six RRR factors were identified. The first RRR factor explained 42.27% of thiamin, 68.34% of riboflavin, 20.92% of niacin, 59.00% of vitamin B6, 56.16% of folate, 41.50% of vitamin B12, and 48.03% of the total variation of six B vitamins. The subsequent five RRR factors explained only 12.73%, 4.71%, 1.67%, 1.45%, and 1.01% of the total variation, separately. Therefore, only the first RRR factor was regarded as the dietary pattern of interest. Since the dietary pattern score was moderately to highly positively correlated with the intakes of six B vitamins (Table 1), this pattern was named the high B-vitamin dietary pattern.

**Figure 1:**
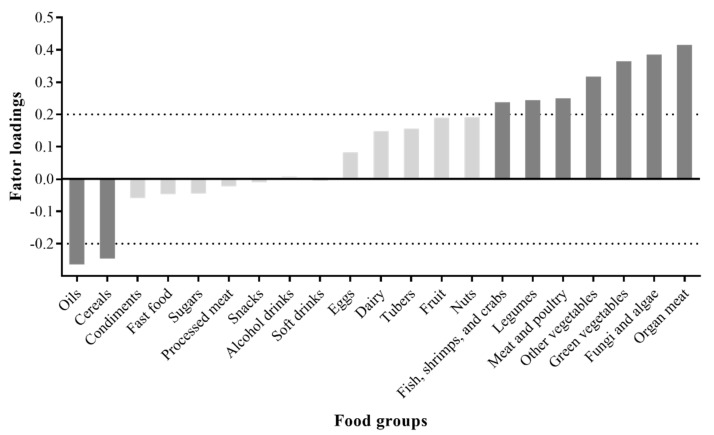
Factor loading of the food groups in the high B-vitamin dietary pattern score. Dark grey indicates absolute factor loading value ≥ 0.2.

The factor loading values of the food groups in the high B-vitamin dietary pattern are shown in Figure 1. The dietary pattern was characterised by high intakes of organ meat; fungi and algae; green vegetables; other vegetables; meat and poultry; legumes; fish, shrimps, and crabs; and low intakes of oils and cereals. As displayed in Table S2, the mean intakes of the selected nutrient varied across quartiles of the high B-vitamin dietary pattern score. On the whole, with the increasing quartiles, women tended to have lower intakes of carbohydrates, the percentage of energy from carbohydrates, potassium, and sodium but higher intakes of protein, the percentage of energy from protein, and most vitamins and minerals.

### 3.2. Participants Characteristics

Maternal socio-demographic characteristics and health-related behaviours differed by quartiles of the high B-vitamin dietary pattern score (Table 2). Women in the highest quartile were more likely to be primiparous, give birth at the ages of 25–29 years, have more than junior school education, drink alcohol, and take iron, calcium, and folic acid supplements. By contrast, women in the highest quartile were less likely to be farmers, be poorer, be exposed to passive smoking, live in rural areas, have pregnancy complications, and use medication.

### 3.3. Dietary Pattern and Birth Outcomes

Associations of the high B-vitamin dietary pattern score with birth outcomes are presented in Table 3 and Table 4. After adjustment for socio-demographic characteristics and health-related behaviours, the 1-SD increase in the high B-vitamin dietary pattern score was associated with 16.4 g (95% CI: 5.4, 27.4 g) higher birth weight, 0.040 (95% CI: 0.014, 0.065) higher birth weight Z score, and 12.1% (OR: 0.879; 95% CI: 0.809, 0.955) lower risk of SGA. Compared with women in the lowest quartile, those in the highest quartile had newborns with a 44.5 g (95% CI: 13.8, 75.2 g; *p*_trend_ = 0.012) higher birth weight, 0.101 (95% CI: 0.029, 0.172; *p*_trend_ = 0.012) higher birth weight Z score, and 27.2% (OR: 0.728; 95% CI: 0.582, 0.910; *p*_trend_ = 0.026) lower risk of SGA.

Stratified analyses according to folic acid and multivitamin supplementation are displayed in Figures S2–S4. A marginally significant interaction was observed between multivitamin supplementation and dietary pattern score on SGA risk (*P*_interaction_ = 0.056). Among the women who did not use multivitamins, per 1-SD increase in the high B-vitamin dietary pattern score was related to 14.2% (OR: 0.858; 95% CI: 0.786, 0.937) lower risk of SGA. In contrast, the association was not significant among users (*p* > 0.05) (Figure S4).

## 4. Discussion

This population-based study conducted in northwest China identified a high B-vitamin dietary pattern, which was characterised by high intakes of animal foods, vegetables, fungi and algae, legumes, and low intakes of oils and cereals. A higher score for the dietary pattern corresponded to higher birth weight and Z score as well as a lower risk of SGA.

To our knowledge, only one other study examined the associations of maternal dietary patterns characterised by B vitamins with neonatal outcomes [39]. In a mother-child cohort in France, 1638 pregnant women before 24 weeks of gestation were recruited to report their diet in the year before pregnancy retrospectively. As compared with our study, the authors regarded one-carbon metabolism nutrients including riboflavin, vitamin B6, folate, vitamin B12, betaine, choline, and methionine as response variables. They also derived a dietary pattern rich in B vitamins, which was loaded positively with low-fat milk, meat, liver, fish, eggs, cereals, mixed vegetables, chicory, leek and cabbage, and broccoli but loaded negatively with snacks and confectionery and sugar-sweetened beverages [39]. The similarity between the dietary patterns in their study and those of ours was that food groups were diverse and balanced. The difference was that our pattern had low intakes of cereals, which may be attributed to the variation in response variables and composition of cereals. The cereals we classified included wheat and rice products, whereas the cereals in their study referred to breakfast cereals and cereal bar fruits. In general, as the bran and germ are removed, commercially available cereals contain small amounts of B vitamins (thiamin, riboflavin, niacin, and folate). Given the protective effects of folic acid on neural tube defects, many countries have created legislation to mandate the fortification of industrially milled cereals with folic acid [40]. However, such projects have not yet been implemented both in China and France. The authors indicated that, when controlling for potential confounders, adherence to the high B-vitamin dietary pattern before pregnancy was not related to birth weight, gestational age, and the risk of SGA [39]. However, in our study, adherence to this dietary pattern during pregnancy was positively associated with birth weight and inversely associated with the risk of SGA. These results may be partly explained by the wide gap in nutritional status between the two populations. The intakes of riboflavin, vitamin B6, folate, and vitamin B12 in our population were approximately 15–65% lower compared with theirs. The benefits of micronutrients were more likely to be observed in malnourished populations. In addition, the timing of interest in the two studies was different. In terms of intrauterine growth, the role of maternal nutrition during pregnancy was far more prominent than that of pre-pregnancy nutrition.

In the present study, it seemed that the high B-vitamins dietary pattern exerted protective effects against SGA only among pregnant women who did not take multivitamins. A plausible explanation for this finding was that multivitamins provided adequate B vitamins to meet the needs of fetal growth. Owing to the variation in the brands of multivitamins, the content of B vitamins in supplements cannot be directly compared with the dietary pattern. Based on a double-blind randomised controlled trial in rural Shaanxi Province, we previously reported that antenatal multiple micronutrients with a recommended allowance of B vitamins resulted in a 44 g increase in birth weight and a 0.19 week increase in gestational age in comparison with folic acid alone [41]. Overall, these studies suggested that prenatal nutrition intervention focusing on B vitamins is probably an effective approach to improve birth outcomes in northwest China.

This study found that neither the continuous nor binary variable of gestational age was relevant to the high B-vitamin dietary pattern. On the contrary, birth weight-related outcomes, including crude birth weight, gestational age- and sex-specific birth weight, and SGA, were all associated with the high B-vitamin dietary pattern. These results suggest that birth weight rather than length of gestation is susceptible to the dietary pattern. Birth weight is the most widely used anthropometric indicator for newborns, in which an increase indicates the promotion of growth and development [42]. In 2012, an estimated 874 000 babies were born SGA in China, with 15 100 attributable neonatal deaths [43]. Babies born SGA are also reported to have a higher risk of delayed neurodevelopment and of being underweight in early adolescence [44,45]. The finding that adherence to the high B-vitamin dietary pattern was associated with a moderate reduction in SGA risk provides one possibility for achieving Sustainable Development Goal 3 (ensuring healthy lives and promoting well-being at all ages). Well-designed longitudinal studies are warranted to validate our findings and to explore the long-term effects of maternal dietary patterns.

Mechanisms that relate antenatal B vitamins and birth weight are still unclear. Almost all B vitamins are involved in one-carbon metabolism and related pathways. Folate is the main carrier of one-carbon units, while riboflavin, vitamin B6, and vitamin B12 act as essential cofactors or precursors of key enzymes [46]. Importantly, one-carbon metabolism plays a role in cellular processes such as biosynthesis, amino acid homeostasis, epigenetic regulation, and redox defence [47]. A growing body of clinical trials have shown that prenatal B vitamins can reduce homocysteine concentrations or alter DNA methylation patterns among newborns [48,49,50], but whether these changes further interfere with birth weight remains to be understood.

It is noteworthy that although our dietary pattern was driven by the variability of B vitamins, the highest quartile was accompanied by the highest intakes of other nutrients, such as protein, vitamin A, vitamin C, calcium, and zinc. We cannot exclude the potential effects of these nutrients on birth weight. Rather than single out individual nutrients that could account for the benefits of the dietary pattern studied, it may be more realistic to explore the synergy of multiple nutrients or food groups.

The present study has some strengths. First, because of the stratified multistage random sampling method, our findings can be generalised to the whole Shaanxi Province. Second, in contrast with previous studies that investigated the role of individual B vitamins in neonatal outcomes, our study explored the beneficial effect of the B vitamins-related dietary pattern using the RRR method, which provides a better foundation for the development of dietary recommendations for pregnant women. Nevertheless, several limitations should be noted. First, temporality and causality cannot be demonstrated from this cross-sectional study. Second, residual confounding cannot be ruled out due to unmeasured or unknown socio-demographic or health-related factors. Third, women were asked to recall pregnancy characteristics within 0–12 months after delivery in our study. Although many studies have shown that pregnancy is a major event during which many features can be recalled well even after years [51,52], the accuracy of our data remains to be validated. Finally, given the convenience and low cost, we evaluated the average dietary intake throughout pregnancy, which was likely to underestimate the importance of maternal diets during a certain stage. Indeed, accumulating evidence indicates that fetal growth is most affected by micronutrient deficiencies at the very earliest embryonic stages [53,54]. Prospective cohort studies that collect maternal dietary intakes over multiple time points from the periconceptional period onwards are required.

## 5. Conclusions

In the cross-sectional study in northwest China, we derived a maternal dietary pattern that was rich in B vitamins using the RRR method and found that greater adherence to this dietary pattern during pregnancy was related to higher birth weight and a lower risk of SGA. Based on these findings, obstetricians should pay more attention to the B-vitamin status of pregnant women and advise women to increase the proportion of animal foods, vegetables, fungi and algae, and legumes in their diets to prevent adverse birth outcomes.

## Figures and Tables

**Table 1:** Associations between the dietary pattern score derived by reduced rank regression and B vitamins intakes.

	*r* (95% CI)	*p*
Thiamin	0.677 (0.664, 0.689)	<0.001
Riboflavin	0.866 (0.860, 0.872)	<0.001
Niacin	0.578 (0.562, 0.593)	<0.001
Vitamin B6	0.809 (0.801, 0.817)	<0.001
Folate	0.841 (0.834, 0.847)	<0.001
Vitamin B12	0.686 (0.674, 0.698)	<0.001

**Table 2:** Maternal characteristics according to quartiles of the high B-vitamin dietary pattern score.

Characteristics	Total	Quartile				*p*_trend_ ^a^
		Q1	Q2	Q3	Q4	
*N*	7347	1837	1837	1837	1836	
Socio-demographic characteristics						
Geographic area (central Shaanxi)	3993 (54.35)	994 (54.11)	1002 (54.55)	956 (52.04)	1041 (56.70)	0.311
Residence (rural)	5612 (76.38)	1560 (84.92)	1530 (83.29)	1395 (75.94)	1127 (61.38)	<0.001
Age at delivery (25–29 years)	2721 (37.35)	650 (35.66)	664 (36.50)	667 (36.51)	740 (40.73)	0.003
Education (more than junior school)	2726 (37.23)	561 (30.62)	577 (31.46)	671 (36.67)	917 (50.19)	<0.001
Occupation (farmer)	5266 (72.21)	1406 (77.00)	1404 (76.93)	1332 (73.11)	1124 (61.76)	<0.001
Household wealth index (poor)	2474 (33.67)	684 (37.23)	686 (37.34)	592 (32.23)	512 (27.89)	<0.001
Parity (primiparous)	4570 (62.22)	1088 (59.23)	1080 (58.82)	1141 (62.15)	1261 (68.68)	<0.001
Health-related behaviours						
Passive smoking	1639 (22.37)	463 (25.23)	430 (23.50)	420 (22.93)	326 (17.81)	<0.001
Alcohol consumption	97 (1.32)	23 (1.25)	15 (0.82)	24 (1.31)	35 (1.91)	0.040
Pregnancy complications	1521 (20.71)	394 (21.46)	405 (22.07)	385 (20.96)	337 (18.37)	0.014
Medication use	1394 (19.02)	380 (20.71)	359 (19.61)	339 (18.49)	316 (17.26)	0.005
Folic acid supplementation	5430 (74.25)	1340 (73.18)	1333 (72.96)	1328 (72.57)	1429 (78.30)	0.001
Calcium supplementation	4796 (65.76)	1172 (64.18)	1194 (65.46)	1170 (63.93)	1260 (69.50)	0.004
Iron supplementation	727 (9.93)	156 (8.51)	171 (9.36)	196 (10.72)	204 (11.15)	0.003
Multivitamin supplementation	647 (8.85)	130 (7.10)	152 (8.33)	165 (9.03)	200 (10.93)	<0.001

Values are *n* (%). ^a^ Obtained from Cochran-Armitage trend tests.

**Table 3:** Associations between the high B-vitamin dietary pattern score and continuous birth outcomes ^a^.

	Continuous ^b^	Quartile				*p*_trend_ ^c^
		Q1	Q2	Q3	Q4	
Birth weight, g ^d^						
Mean ± SD	3270.2 ± 448.1	3244.4 ± 452.0	3269.5 ± 451.5	3259.5 ± 451.0	3307.4 ± 435.6	
Model 1 ^e^	18.1 (7.5, 28.7)	Ref	26.2 (−2.8, 55.3)	12.5 (−16.8, 41.8)	52.3 (22.5, 82.2)	0.002
Model 2 ^f^	16.3 (5.5, 27.1)	Ref	25.3 (−3.9, 54.6)	8.3 (−21.2, 37.8)	45.4 (15.0, 75.7)	0.010
Model 3 ^g^	16.4 (5.4, 27.4)	Ref	21.9 (−7.6, 51.5)	6.4 (−23.4, 36.1)	44.5 (13.8, 75.2)	0.012
Gestational age at birth, weeks						
Mean ± SD	39.54 ± 1.50	39.55 ± 1.32	39.55 ± 1.38	39.55 ± 1.60	39.53 ± 1.69	
Model 1 ^e^	0.002 (−0.033, 0.038)	Ref	−0.020 (−0.116, 0.077)	−0.003 (−0.101, 0.094)	0.010 (−0.090, 0.109)	0.785
Model 2 ^f^	0.002 (−0.034, 0.038)	Ref	−0.011 (−0.108, 0.086)	−0.002 (−0.100, 0.096)	0.012 (−0.089, 0.114)	0.777
Model 3 ^g^	−0.001 (−0.037, 0.037)	Ref	−0.006 (−0.105, 0.092)	−0.004 (−0.104, 0.095)	0.008 (−0.095, 0.111)	0.870
Birth weight Z score						
Mean ± SD	−0.03 ± 1.04	−0.09 ± 1.05	−0.03 ± 1.04	−0.05 ± 1.07	0.07 ± 1.01	
Model 1 ^e^	0.042 (0.017, 0.067)	Ref	0.073 (0.005, 0.140)	0.032 (−0.036, 0.100)	0.116 (0.047, 0.186)	0.002
Model 2 ^f^	0.039 (0.014, 0.064)	Ref	0.069 (0.002, 0.137)	0.023 (−0.045, 0.091)	0.101 (0.030, 0.171)	0.010
Model 3 ^g^	0.040 (0.014, 0.065)	Ref	0.059 (−0.009, 0.128)	0.019 (−0.050, 0.088)	0.101 (0.029, 0.172)	0.012

Ref, reference. ^a^ Two-level generalised linear mixed models were used to estimate mean differences and 95% CIs. ^b^ Per 1-SD increase in the high B-vitamin dietary pattern score. ^c^ Obtained using the median value of each dietary pattern quartile as a continuous variable in the regression models. ^d^ Precise to 10 g. ^e^ Unadjusted. ^f^ Adjusted for socio-demographic characteristics, including geographic area, residence, age at delivery, education, occupation, household wealth index and parity. ^g^ Adjusted for all variables in model 2 plus health-related behaviours, including passive smoking, alcohol consumption, pregnancy complications, medication use, as well as iron, calcium, folic acid, and multivitamin supplementation.

**Table 4:** Associations between the high B-vitamin dietary pattern score and dichotomous birth outcomes ^a^.

	Continuous ^b^	Quartile				*p*_trend_ ^c^
		Q1	Q2	Q3	Q4	
LBW						
*n* (*%*)	226 (3.10)	58 (3.20)	59 (3.24)	64 (3.51)	45 (2.47)	
Model 1 ^d^	0.901 (0.782, 1.039)	Ref	1.018 (0.704, 1.473)	1.111 (0.773, 1.598)	0.771 (0.518, 1.149)	0.256
Model 2 ^e^	0.909 (0.786, 1.052)	Ref	1.004 (0.693, 1.455)	1.129 (0.784, 1.626)	0.796 (0.529, 1.196)	0.375
Model 3 ^f^	0.923 (0.797, 1.069)	Ref	1.009 (0.695, 1.465)	1.125 (0.780, 1.623)	0.833 (0.553, 1.255)	0.501
Preterm						
*n* (*%*)	227 (3.09)	60 (3.27)	64 (3.49)	57 (3.11)	46 (2.51)	
Model 1 ^d^	0.940 (0.817, 1.081)	Ref	1.077 (0.751, 1.544)	0.925 (0.637, 1.344)	0.718 (0.481, 1.072)	0.077
Model 2 ^e^	0.940 (0.815, 1.086)	Ref	1.065 (0.739, 1.534)	0.895 (0.613, 1.307)	0.699 (0.464, 1.053)	0.062
Model 3 ^f^	0.944 (0.817, 1.092)	Ref	1.042 (0.721, 1.506)	0.885 (0.604, 1.295)	0.701 (0.463, 1.061)	0.070
SGA						
*n* (*%*)	843 (11.54)	244 (13.33)	205 (11.22)	234 (12.81)	160 (8.79)	
Model 1 ^d^	0.852 (0.786, 0.924)	Ref	0.818 (0.669, 0.999)	0.977 (0.803, 1.189)	0.660 (0.532, 0.821)	0.001
Model 2 ^e^	0.874 (0.805, 0.948)	Ref	0.811 (0.662, 0.992)	0.988 (0.810, 1.204)	0.705 (0.566, 0.879)	0.011
Model 3 ^f^	0.879 (0.809, 0.955)	Ref	0.831 (0.678, 1.020)	1.016 (0.832, 1.241)	0.728 (0.582, 0.910)	0.026

LBW, low birth weight; Ref, reference; SGA, small-for-gestational-age. ^a^ Two-level generalised linear mixed models were used to estimate ORs and 95% CIs. ^b^ Per 1-SD increase in the high B-vitamin dietary pattern score. ^c^ Obtained using the median value of each dietary pattern quartile as a continuous variable in the regression models. ^d^ Unadjusted. ^e^ Adjusted for socio-demographic characteristics, including geographic area, residence, age at delivery, education, occupation, household wealth index and parity. ^f^ Adjusted for all variables in model 2 plus health-related behaviours, including passive smoking, alcohol consumption, pregnancy complications, medication use, as well as iron, calcium, folic acid, and multivitamin supplementation.

## Supplementary Materials

The following supporting information can be downloaded at: https://www.mdpi.com/article/10.3390/nu14030600/s1, Table S1: Foods items within each food group; Table S2: Selected nutrient intakes according to quartiles of the high B-vitamin dietary pattern score; Figure S1: Flow chart of the selection of study participants; Figure S2: Associations of 1-SD increase in the high B-vitamin dietary pattern score with birth weight stratified by the use of B vitamins-containing supplements; Figure S3: Associations of 1-SD increase in the high B-vitamin dietary pattern score with birth weight Z score stratified by the use of B vitamins-containing supplements; Figure S4: Associations of 1-SD increase in the high B-vitamin dietary pattern score with SGA stratified by the use of B vitamins-containing supplements.
