# Supplementary material for: Associations of B Vitamin-Related Dietary Pattern during Pregnancy with Birth Outcomes: A Population-Based Study in Northwest China
Source: Nutrients. 2022 Jan 29;14(3):600. doi: 10.3390/nu14030600 (PMC8838836; doi:10.3390/nu14030600)
Supplement: Supplementary file 1 [file nutrients-14-00600-s001.zip › nutrients-1551410-supplementary.pdf]

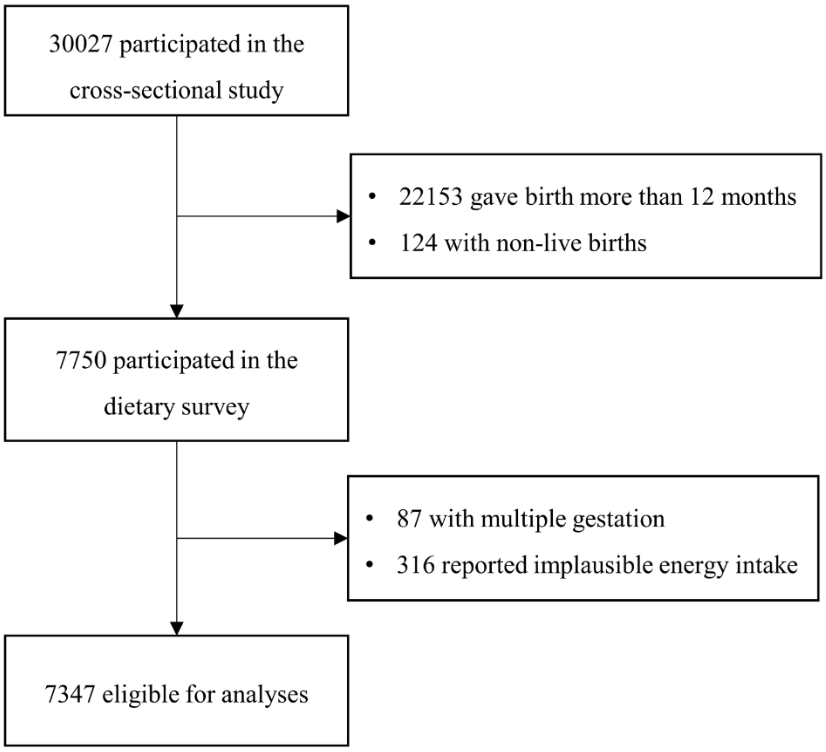

Supplemental Figure S1. Flow chart of the selection of study participants.

Supplemental Table S1. Foods items within each food group.

Food groups	Food items
Cereals	Wheat noodle, wheat bun, pancake, steamed rice, vegetarian dumpling, meat dumpling, rice noodle, rice porridge, wheat flour
Green vegetables	Kidney bean, green pepper, cucumber, balsam pear, zucchini, Chinese chive, cabbage, broccoli, spinach, water spinach, Chinese celery stem, romaine lettuce, chrysanthemum crown daisy, lettuce stem, bamboo shoot, spring onion
Other vegetables	Radish, carrot, bean sprout, tomato, Chinese wax gourd, pumpkin, cauliflower, lotus root, onion, garlic bulb
Tubers	Potato, sweet potato, yam
Fruit	Apple, orange, banana, plum, pineapple, cherry, shaddock, watermelon, melon, jujube, pear, grape, persimmon
Legumes	Soybean, mung bean, tofu, soybean curd, soybean milk, soybean curd slab
Eggs	Egg
Dairy	Milk, powdered milk, yoghurt
Meat and poultry	Pork, beef, mutton, chicken
Organ meat	Liver, blood, kidney, others
Processed meat	Cured pork, cured sausage, ham sausage
Fish, shrimps, and crabs	Freshwater fish, saltwater fish, dried shrimp, fresh shrimp or crab
Fungi and algae	Enoki mushroom, shiitake mushroom, oyster mushroom, wood ear fungi, kelp, laver
Nuts	Walnut, peanut, sunflower seed
Fast food	Instant noodles, bread, biscuit
Snacks	Starch jelly sheet, ice cream, rice crust, mungbean cake, cake
Soft drinks	Tea, coffee, soda drink, juice
Alcohol drinks	Beer, Chinese Baijiu, wine
Sugars	Toffee, chocolate, sugar
Oils	Lard, rapeseed oil
Condiments	Salt, tamari

Supplemental Table S2. Selected nutrient intakes according to quartiles of the high B-vitamin dietary pattern score.

Nutrients	Total	Quartile				P_{trend}
		Q1	Q2	Q3	Q4	
Total energy (kcal)	2322.90 ± 781.00	2540.49 ± 729.19	2155.52 ± 667.36	2113.36 ± 725.57	2482.31 ± 890.29	0.008
% Energy from protein	11.87 ± 2.16	10.52 ± 1.65	11.28 ± 1.61	11.91 ± 1.71	13.77 ± 2.17	<0.001
% Energy from fat	34.17 ± 8.81	35.21 ± 9.88	33.26 ± 8.65	33.53 ± 8.51	34.70 ± 7.95	0.172
% Energy from carbohydrates	59.57 ± 9.43	59.38 ± 10.02	60.95 ± 8.90	60.27 ± 9.11	57.67 ± 9.35	<0.001
Protein (g)	70.42 ± 30.98	56.19 ± 9.32	64.18 ± 7.05	67.81 ± 7.98	77.09 ± 13.50	<0.001
Fat (g)	87.84 ± 37.10	85.82 ± 27.23	80.94 ± 20.61	82.06 ± 19.63	85.54 ± 21.70	0.908
Carbohydrates (g)	345.24 ± 124.41	331.33 ± 64.47	336.87 ± 49.22	331.56 ± 48.22	317.03 ± 58.20	<0.001
Vitamin A (µg, RAE)	573.80 ± 729.93	211.21 ± 293.36	392.78 ± 244.22	516.86 ± 317.69	984.35 ± 1060.2	<0.001
Thiamin (mg)	0.73 ± 0.33	0.56 ± 0.13	0.67 ± 0.10	0.72 ± 0.10	0.81 ± 0.15	<0.001
Riboflavin (mg)	0.79 ± 0.50	0.47 ± 0.18	0.65 ± 0.14	0.77 ± 0.16	1.07 ± 0.39	<0.001
Niacin (mg)	15.39 ± 7.24	12.74 ± 3.74	13.91 ± 3.27	14.44 ± 3.28	17.15 ± 5.50	<0.001
Vitamin B6 (mg)	1.41 ± 0.71	0.95 ± 0.27	1.22 ± 0.22	1.40 ± 0.24	1.75 ± 0.41	<0.001
Folate (µg)	322.18 ± 210.28	181.03 ± 87.19	263.32 ± 74.51	313.92 ± 84.18	441.34 ± 159.61	<0.001
Vitamin B12 (µg)	3.69 ± 4.02	1.48 ± 1.79	2.67 ± 1.70	3.42 ± 2.00	5.94 ± 5.06	<0.001
Vitamin C (mg)	106.95 ± 76.81	63.53 ± 51.08	89.92 ± 45.11	108.46 ± 50.31	139.67 ± 71.29	<0.001
Vitamin E (mg)	41.64 ± 17.84	40.04 ± 14.71	38.40 ± 12.33	39.09 ± 12.09	42.28 ± 14.31	<0.001
Calcium (mg)	616.52 ± 335.88	440.94 ± 174.35	549.55 ± 155.27	609.28 ± 162.49	715.07 ± 236.93	<0.001
Iron (mg)	31.74 ± 15.38	30.18 ± 13.70	30.39 ± 11.01	29.09 ± 9.95	31.40 ± 10.79	0.051
Zinc (mg)	6.77 ± 3.73	4.50 ± 1.21	5.76 ± 0.96	6.59 ± 1.03	8.41 ± 1.97	<0.001
Potassium (mg)	2481.25 ± 1234.00	2540.49 ± 729.19	2155.52 ± 667.36	2113.36 ± 725.57	2482.31 ± 890.29	0.008
Sodium (mg)	4254.16 ± 2117.46	4340.32±2323.17	4147.68±1811.99	4083.69±1909.77	4034.71±1881.40	<0.001

RAE, retinol activity equivalent.

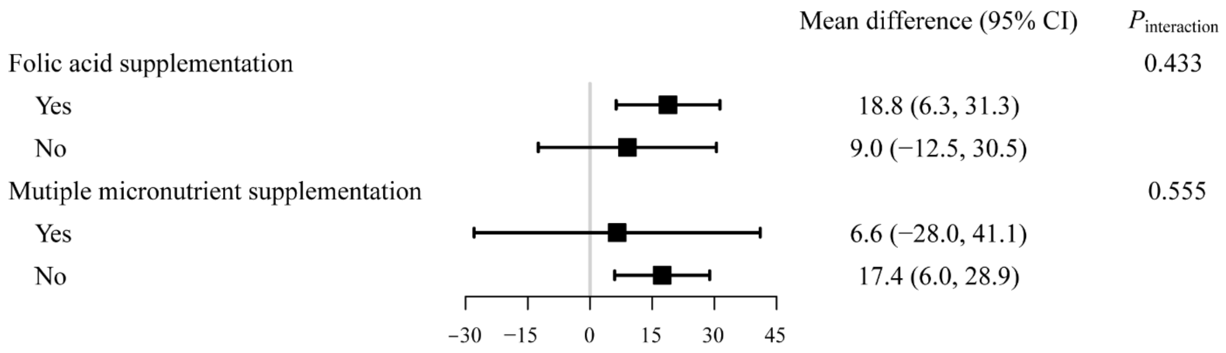

Supplemental Figure S2. Associations of 1-SD increase in the high B-vitamin dietary pattern score with birth weight stratified by the use of B vitamins-containing supplements. Mean differences and 95% CIs were estimated by using two-level generalised linear mixed models and adjusted for the geographic area, residence, age at delivery, education, occupation, household wealth index, parity, passive smoking, alcohol consumption, pregnancy complications, medication use, as well as iron, calcium, folic acid, and multiple micronutrient supplementation.

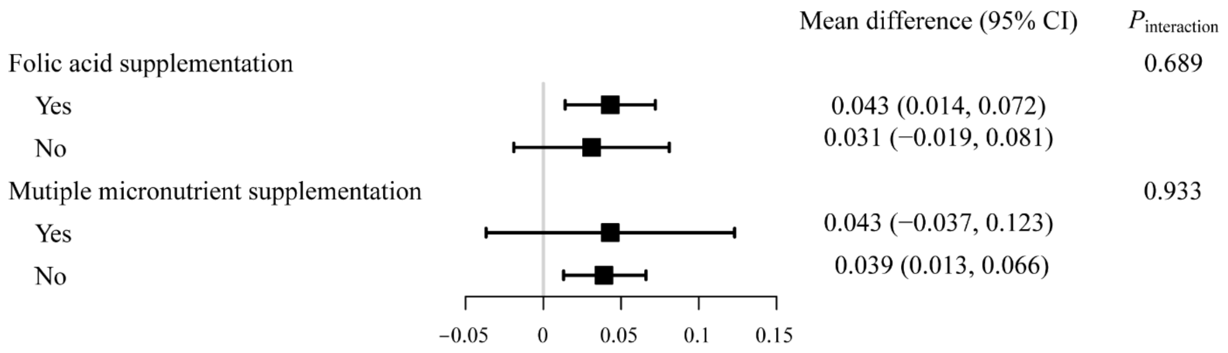

Supplemental Figure S3. Associations of 1-SD increase in the high B-vitamin dietary pattern score with birth weight Z score stratified by the use of B vitamins-containing supplements. Mean differences and 95% CIs were estimated by using two-level generalised linear mixed models and adjusted for the geographic area, residence, age at delivery, education, occupation, household wealth index, parity, passive smoking, alcohol consumption, pregnancy complications, medication use, as well as iron, calcium, folic acid, and multiple micronutrient supplementation.

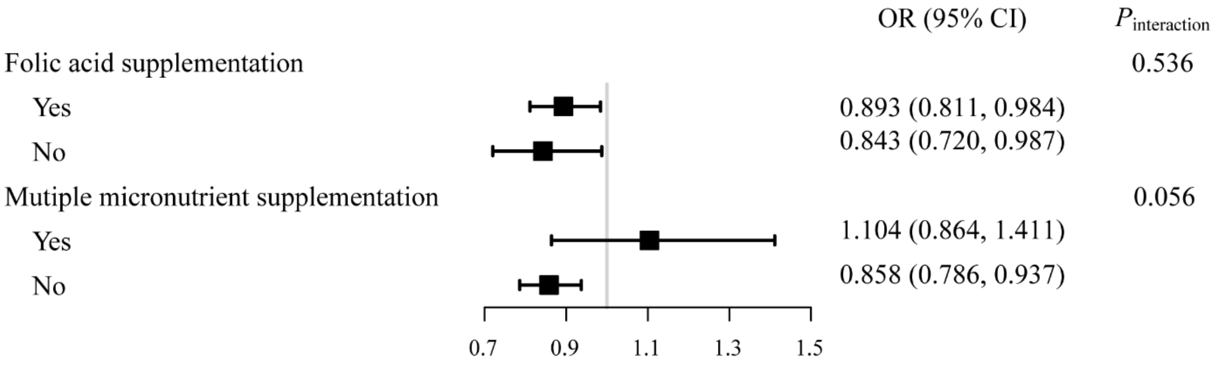

Supplemental Figure S4. Associations of 1-SD increase in the high B-vitamin dietary pattern score with SGA stratified by the use of B vitamins-containing supplements. ORs and 95% CIs were estimated by using two-level generalised linear mixed models and adjusted for the geographic area, residence, age at delivery, education, occupation, household wealth index, parity, passive smoking, alcohol consumption, pregnancy complications, medication use, as well as iron, calcium, folic acid, and multiple micronutrient supplementation.
